# Kinase activity of SOBIR1 and BAK1 is required for immune signalling

**DOI:** 10.1111/mpp.12767

**Published:** 2019-01-02

**Authors:** Aranka M. van der Burgh, Jelle Postma, Silke Robatzek, Matthieu H. A. J. Joosten

**Affiliations:** ^1^ Laboratory of Phytopathology Wageningen University Droevendaalsesteeg 1 6708 PB Wageningen the Netherlands; ^2^ The Sainsbury Laboratory Norwich Research Park, Norwich NR4 7UH UK; ^3^ Ludwig‐Maximilians‐Universität München Genetics Großhaderner Str. 2–4 82152 Martinsried Germany

**Keywords:** BAK1/SERK3, Cf‐4, immunity, phosphorylation, RLK, RLP, SOBIR1

## Abstract

Leucine‐rich repeat‐receptor‐like proteins (LRR‐RLPs) and LRR‐receptor‐like kinases (LRR‐RLKs) trigger immune signalling to promote plant resistance against pathogens. LRR‐RLPs lack an intracellular kinase domain, and several of these receptors have been shown to constitutively interact with the LRR‐RLK Suppressor of BIR1‐1/EVERSHED (SOBIR1/EVR) to form signalling‐competent receptor complexes. Ligand perception by LRR‐RLPs initiates recruitment of the co‐receptor BRI1‐Associated Kinase 1/Somatic Embryogenesis Receptor Kinase 3 (BAK1/SERK3) to the LRR‐RLP/SOBIR1 complex, thereby activating LRR‐RLP‐mediated immunity. We employed phosphorylation analysis of *in planta*‐produced proteins, live cell imaging, gene silencing and co‐immunoprecipitation to investigate the roles of SOBIR1 and BAK1 in immune signalling. We show that *Arabidopsis thaliana* (*At*) SOBIR1, which constitutively activates immune responses when overexpressed *in planta*, is highly phosphorylated*.* Moreover, in addition to the kinase activity of SOBIR1 itself, kinase‐active BAK1 is essential for *At*SOBIR1‐induced constitutive immunity and for the phosphorylation of *At*SOBIR1. Furthermore, the defence response triggered by the tomato LRR‐RLP Cf‐4 on perception of Avr4 from the extracellular pathogenic fungus *Cladosporium fulvum* is dependent on kinase‐active BAK1. We argue that, in addition to the trans‐autophosphorylation of SOBIR1, it is likely that SOBIR1 and BAK1 transphosphorylate, and thereby activate the receptor complex. The signalling‐competent cell surface receptor complex subsequently activates downstream cytoplasmic signalling partners to initiate RLP‐mediated immunity.

## Introduction

Plants are sessile organisms and possess several layers of defence to protect themselves against pathogens. One layer comprises transmembrane (TM) receptors which are present at the cell surface. Unlike mammals, plants have evolved highly expanded families of TM receptors, which sense non‐self or danger‐related molecules in the extracellular space and initiate downstream signalling to mount plant immunity (Zipfel, 2014). TM receptors comprise receptor‐like kinases (RLKs) and receptor‐like proteins (RLPs). Both RLKs and RLPs contain ectodomains for ligand recognition, which, in many cases, are leucine‐rich repeat (LRR) domains (Böhm *et al.*, 2014; Couto and Zipfel, 2016; Macho and Zipfel, 2014). LRR‐RLKs (further referred to as RLKs) carry an intracellular kinase domain, whereas LRR‐RLPs (further referred to as RLPs) do not. Whilst the expansion of receptor families enables co‐evolution with infectious pathogens, it challenges plants to link newly evolved receptors with downstream immune signalling pathways. In line with this, it is now emerging that receptors form heteromeric kinase complexes to induce immune signalling (Dufayard *et al.*, 2017; Fischer *et al.*, 2016).

RLPs, lacking an intracellular signalling domain, constitutively interact with the RLK Suppressor Of BIR1‐1/EVERSHED (SOBIR1/EVR, hereafter referred to as SOBIR1), thereby providing a kinase domain that is thought to function in downstream signalling (Gao *et al.*, 2009; Gust and Felix, 2014; Leslie *et al.*, 2010; Liebrand *et al.*, 2014). SOBIR1 was initially found to interact with Cf proteins from tomato (*Solanum lycopersicum*, *Sl*), conferring resistance to the extracellular fungal pathogen *Cladosporium fulvum *(Liebrand *et al.*, 2013). Since this initial discovery, it has been shown that SOBIR1 constitutively interacts with many RLPs involved in immunity and development (Bi *et al.*, 2014; Catanzariti *et al.*, 2017; Domazakis *et al*., 2018; Hegenauer *et al.*, 2016; Jehle *et al.*, 2013; Liebrand *et al.*, 2013; Ma and Borhan, 2015; Wang *et al.*, 2018; Zhang *et al.*, 2014; Zhang *et al.*, 2013a). In addition to the stabilization of the associated RLP by SOBIR1, SOBIR1 is thought to be involved in downstream signalling on RLP activation by its matching ligand (Liebrand *et al.*, 2013, 2014).

The RLK BRI1‐Associated Receptor Kinase 1/Somatic Embryogenesis Receptor Kinase 3 (BAK1/SERK3, hereafter referred to as BAK1) functions as a co‐receptor of several well‐studied RLKs from *Arabidopsis*, including Flagellin‐Sensing 2 (FLS2), Elongation Factor‐Tu Receptor (EFR) and the brassinosteroid (BR) receptor Brassinosteroid‐Insensitive 1 (BRI1) (Gómez‐Gómez and Boller, 2000; Li and Chory, 1997; Nam and Li, 2002; Zipfel *et al.*, 2006). These RLKs form complexes with BAK1 on association with their ligands (Chinchilla *et al.*, 2007; Heese *et al.*, 2007; Santiago *et al.*, 2013; Somssich *et al.*, 2015; Sun *et al.*, 2013a, 2013b). RLK/BAK1 complex formation is followed by the transphosphorylation of both kinase domains, the initiation of downstream signalling and the internalization of the activated RLK/BAK1 complex through endocytosis (Couto and Zipfel, 2016; Frescatada‐Rosa *et al.*, 2015; Schwessinger *et al.*, 2011; Wang *et al.*, 2008). Differential auto‐ and transphosphorylation have been suggested to take place for BAK1 when complexed with RLKs, signalling for either defence or development, and this differential phosphorylation eventually leads to the desired output (Macho *et al.*, 2014, 2015; Oh *et al.*, 2011; Schwessinger *et al.*, 2011; Wang *et al.*, 2008).

Similar to the situation with RLKs, BAK1 has also recently been found to be recruited to two‐component RLP/SOBIR1 complexes on ligand recognition by the RLP involved (Albert *et al.*, 2015; Domazakis *et al*., 2018; Postma *et al.*, 2016; Wang *et al.*, 2018). For example, BAK1 is specifically recruited to the Cf‐4/SOBIR1 complex on perception of Avr4 from *C. fulvum* by Cf‐4 (Postma *et al.*, 2016). Similarly, Albert *et al*. (2015) showed that the RLP23/SOBIR1 complex recruits BAK1 to mediate immunity triggered by necrosis and ethylene‐inducing peptide‐like 1 proteins (NLPs). Reminiscent of BAK1‐mediated receptor complex activation for cell surface complexes involving the RLKs FLS2, EFR and BRI1, it is likely that BAK1, on recruitment, also forms signalling‐competent receptor complexes with RLP/SOBIR1 bipartite RLKs, thereby mediating RLP signalling. In agreement with this, SOBIR1 kinase activity is essential for its function downstream of Cf‐4, as it has been shown that a kinase‐dead mutant of SOBIR1 is unable to complement Cf‐4/Avr4 signalling and endocytosis of the Cf‐4/SOBIR1 complex (Bi *et al.*, 2015; Liebrand *et al.*, 2013; Postma *et al.*, 2016). However, until now, it has remained unclear how the kinase domain of SOBIR1 contributes to RLP/SOBIR1 receptor complex signalling.

SOBIR1 was initially identified as a positive regulator of cell death. It was found that *Arabidopsis BAK1‐Interacting RLK 1 *(*bir1‐1*) loss‐of‐function mutants showed severe dwarfing as a result of constitutive immunity, which was partially suppressed by a *sobir1* loss‐of‐function mutation (Gao *et al.*, 2009). Furthermore, overexpression of SOBIR1 in *Arabidopsis* induced constitutive immunity, which was observed as constitutive cell death and defence gene activation (Gao *et al.*, 2009). BIR1 is a negative regulator of defence, which sequesters BAK1 away from active signalling complexes (Gao *et al.*, 2009; Liu *et al.*, 2016). Liu *et al.* (2016) showed that, upon silencing of *BIR1* in *Arabidopsis*, more BAK1 becomes available, and constitutively interacts with SOBIR1.

Recently, we have observed that transient heterologous overexpression of *Arabidopsis thaliana* (*At*)SOBIR1 in *Nicotiana tabacum* (tobacco, *Nt*) and *N. benthamiana *(*Nb*), in contrast with transient overexpression of *Sl*SOBIR1 and *Nb*SOBIR1, also results in constitutive immunity (Wu *et al.*, 2018). This constitutive immunity is typically manifested by the induction of cell death [the hypersensitive response (HR)] and mitogen‐activated protein kinase (MAPK) activation (Wu *et al.*, 2018). Kinase‐dead *At*SOBIR1^D498N ^does not induce constitutive immunity, indicating that SOBIR1 kinase activity is essential for this phenomenon. The lack of constitutive immune activation by *Sl*SOBIR1 and *Nb*SOBIR1 in tobacco and *N. benthamiana* might be explained by the negative regulation of these Solanaceous orthologues of SOBIR1 by endogenous phosphatases of the Solanaceous plants tobacco and *N. benthamiana*. Negative regulation of immune receptors via dephosphorylation is a well‐known phenomenon (Couto and Zipfel, 2016). BAK1, for example, has been shown to be negatively regulated by Protein Phosphatase type 2A (PP2A) (Segonzac *et al.*, 2014). Possibly, endogenous Solanaceous phosphatases successfully negatively regulate the activity of tomato and *N. benthamiana* SOBIR1 via dephosphorylation. However, these phosphatases might not be able to keep *At*SOBIR1 in check, for example, because of the lower affinity for this heterologous SOBIR1 orthologue (Wu *et al.*, 2018).

Here, we set out to determine how SOBIR1 activates defence signalling. For this, we exploited the phenomenon of *At*SOBIR1 triggering constitutive immune signalling in *N. benthamiana*, leading to cell death. We show that *At*SOBIR1 is clearly phosphorylated when transiently expressed in *N. benthamiana*, whereas the kinase‐dead mutant *At*SOBIR1^D498N^ is not. We demonstrate that the overall phosphorylation status of *At*SOBIR1 is positively linked with its constitutive immune activity. We found that SOBIR1 constitutively forms homodimers, as well as heterodimers, with BAK1. Interestingly, the Cf‐4/Avr4‐triggered defence response, as well as *At*SOBIR1 constitutive immunity and *At*SOBIR1 phosphorylation, all depend on defence signalling‐competent BAK1, in addition to SOBIR1 kinase activity. These findings are in agreement with a model in which BAK1, which is recruited to the RLP/SOBIR1 complex on ligand perception by an RLP, and SOBIR1 probably transphosphorylate each other to signal for immunity.

## Results

### Constitutive immune activity of *At*SOBIR1 is positively linked with its phosphorylation status

**Figure 1:**
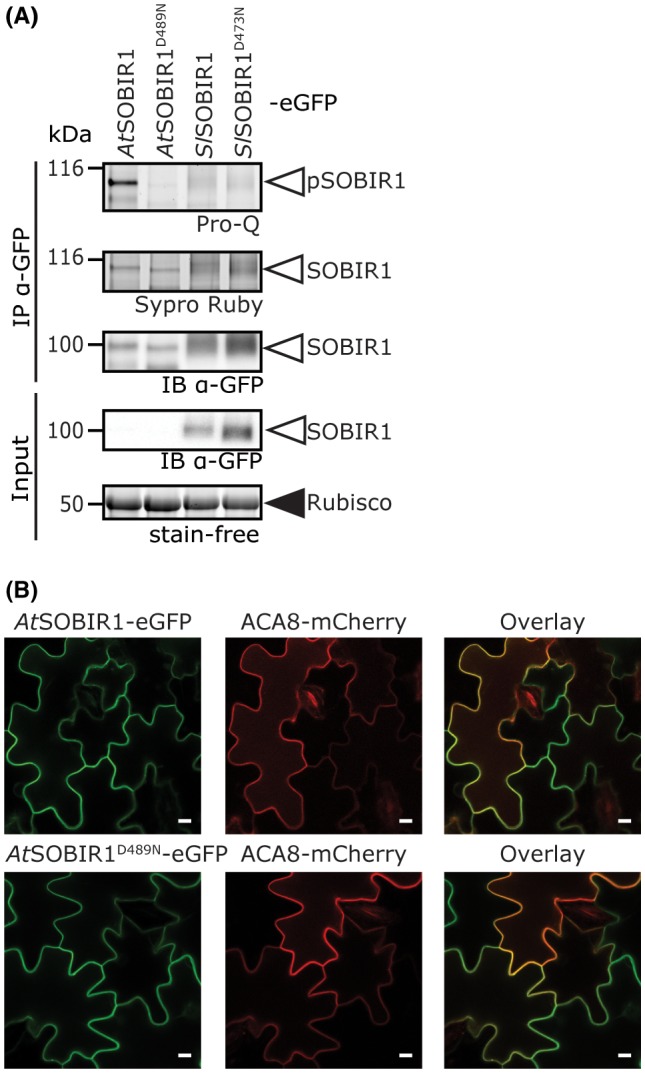
Constitutive immune activity of *Arabidopsis thaliana* (*At*)SOBIR1 positively links with its phosphorylation status. (A) Pro‐Q phosphoprotein staining of immunoprecipitated enhanced green fluorescent protein (eGFP)‐tagged SOBIR1 variants from *Arabidopsis* and tomato shows constitutive phosphorylation of *At*SOBIR1 (p*At*SOBIR1), but not of *Solanum lycopersicum* (*Sl*)SOBIR1. Furthermore, both the *Arabidopsis* and tomato kinase‐dead mutants show no obvious phosphorylation. Agroinfiltrations were performed on *Nicotiana benthamiana *leaves at an optical density at 600 nm (OD_600_) of 1, with co‐infiltration of P19 also at an OD_600_ of 1. Leaves were harvested about 40 h after agroinfiltration, before cell death became apparent, followed by immunoprecipitation (IP) using anti‐GFP affinity beads. Sypro Ruby staining and immune blotting (IB) show SOBIR1 protein levels. It should be noted that the band representing p*At*SOBIR1 shows a slightly higher molecular weight than unphosphorylated *At*SOBIR1^D498N^ on the Sypro Ruby‐stained gel and αGFP IB. The ribulose‐1,5‐bisphosphate carboxylase/oxygenase (Rubisco) band of the input shows equal loading. (See also Fig. S1.) It should be noted that, because of its low level of accumulation, *At*SOBIR1 is not visible in the input sample. (B) *At*SOBIR1 and its kinase‐dead version localize at the plasma membrane. Leaves of tobacco were agroinfiltrated with constructs driving the expression of eGFP‐tagged wild‐type *At*SOBIR1 or the kinase‐dead mutant *At*SOBIR1^D489N^, and plasma membrane‐localized ACA8‐mCherry was co‐expressed. Overlay images indicate the co‐localization of the proteins fused to GFP and mCherry, as a yellow colour is produced (right panels). Confocal microscopy analysis was performed at 2 days post‐infiltration (dpi). White bars represent 10 µm. The experiment was performed three times and representative photographs are shown.

Leslie *et al*. (2010) showed that the kinase domain of *At*SOBIR1 trans‐autophosphorylates *in vitro* at serine (Ser), threonine (Thr) and tyrosine (Tyr) residues. To determine whether *in planta *phosphorylation of SOBIR1 plays a role in signalling for constitutive immunity induced by this RLK (Wu *et al.*, 2018), we transiently overexpressed full‐length enhanced green fluorescent protein (eGFP)‐tagged *Arabidopsis* and tomato SOBIR1 proteins, and the corresponding kinase‐dead mutants in which the catalytic aspartic acid (Asp, D) is mutated to asparagine (Asn, N), in *N. benthamiana* in combination with P19. Subsequently, we analysed their overall phosphorylation status by Pro‐Q staining. This revealed that *At*SOBIR1 is highly phosphorylated, whereas *Sl*SOBIR1 and both kinase‐dead mutants are not (Fig. 1A). It appears that the phosphorylation status of *At*SOBIR1 is positively linked with its constitutive immune activity (Fig. S1A, see Supporting Information) (Wu *et al.*, 2018). The lack of a Pro‐Q signal for kinase‐dead *At*SOBIR1 suggests that the wild‐type RD‐kinase of *At*SOBIR1 trans‐autophosphorylates *in planta*, as it has previously been shown to do *in vitro* (Leslie *et al.*, 2010). In addition, the low phosphorylation status of *At*SOBIR1^D489N^ suggests that it might be necessary for *At*SOBIR1 to first activate a potential signalling partner, which then, in its turn, fully activates *At*SOBIR1 by transphosphorylation. Observations by confocal microscopy showed that, similar to previous observations for *Sl*SOBIR1 (Postma *et al.*, 2016), both wild‐type *At*SOBIR1‐eGFP and kinase‐dead *At*SOBIR1^R489N^‐eGFP localize at the plasma membrane (PM), where such events can take place (Fig. 1B).

Interestingly, constitutive immunity and the functionality of SOBIR1 downstream of Cf‐4 are dependent on SOBIR1 kinase activity (Liebrand *et al.*, 2013; Wu *et al.*, 2018). The stabilization of Cf‐4 by SOBIR1 is independent of SOBIR1 kinase activity, and so this stabilization effect by itself cannot explain the role of SOBIR1 in RLP‐mediated signalling (Fig. S1B) (Liebrand *et al.*, 2013). This again points to a signalling role by the kinase domain of SOBIR1. The lack of a Pro‐Q signal for *Sl*SOBIR1 supports the hypothesis that negative regulation probably takes place through dephosphorylation of this solanaceous SOBIR1 by endogenous phosphatases in *N. benthamiana* (Fig. 1A), as suggested by Wu *et al.* (2018) and reviewed by Couto and Zipfel (2016).

Together, these data show that the constitutive immune activity of *At*SOBIR1 is positively linked with its phosphorylation status, and that the immune activity of *Sl*SOBIR1 is probably kept in check by maintaining low phosphorylation levels.

### SOBIR1 constitutively forms homodimers

**Figure 2:**
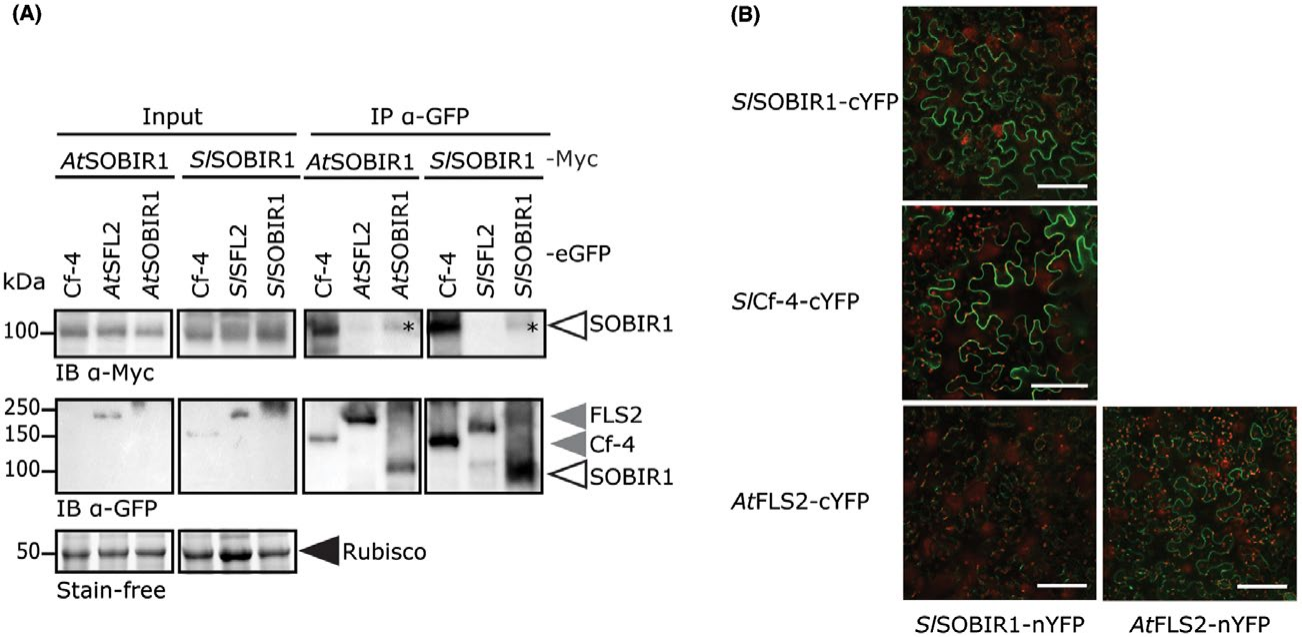
SOBIR1 constitutively forms homodimers *in planta*. (A) Myc‐tagged versions of *At*SOBIR1 and *Sl*SOBIR1 co‐immunoprecipitate with eGFP‐tagged versions of *At*SOBIR1 and *Sl*SOBIR1 (asterisks), respectively, and with Cf‐4‐eGFP, but not with Flagellin‐Sensing 2 (FLS2)‐eGFP. Co‐agroinfiltrations of the various affinity‐tagged proteins were performed in combination with P19 in *N. benthamiana* leaves at an optical density at OD_600_ of 0.6 for each construct. Leaves were harvested at 2 dpi, and subjected to IP using anti‐GFP beads, followed by immune blotting (IB). The Rubisco band of the input shows equal loading. The experiment was performed twice and representative results are shown. It should be noted that, because of the low accumulation levels, not all proteins are visible in the input samples. (B) A split‐YFP (yellow fluorescent protein) experiment shows the interaction between *Sl*SOBIR1‐nYFP and *Sl*SOBIR1‐cYFP at the plasma membrane. Leaves of *N. benthamiana* were agroinfiltrated with constructs driving expression of the indicated constructs, and analysed for interaction at 2 dpi using confocal microscopy. Chloroplast autofluorescence is depicted in red. White bars represent 100 µm. The experiment was performed three times and representative photographs are shown. [Colour figure can be viewed at wileyonlinelibrary.com]

To explore whether trans‐autophosphorylation of SOBIR1 might play a role in signalling for defence by the RD‐kinase SOBIR1, we analysed whether SOBIR1 forms homodimers *in planta*. Transient co‐expression of eGFP‐ and Myc‐tagged SOBIR1 orthologues, followed by immunoprecipitation (IP) of eGFP‐tagged SOBIR1, resulted in the co‐purification of SOBIR1‐Myc (Fig. 2A). This indicates that both *At*SOBIR1 and *Sl*SOBIR1 form homodimers *in planta*. In addition, kinase‐dead variants of *At*SOBIR1 and *Sl*SOBIR1 also form homodimers (Fig. S2, see Supporting Information). This shows that the lack of phosphorylation by kinase‐dead *At*SOBIR1^D489N^ is not caused by an inability to form homodimers. Much higher amounts of SOBIR1‐Myc co‐purified with the IP of Cf‐4‐eGFP, used as a positive control, than with the IP of SOBIR1‐eGFP (Fig. 2A). This suggests that probably only a small pool of SOBIR1 protein is present in the form of homodimers *in planta*, and this probably explains why the homodimerization of SOBIR1 was not observed previously (Liebrand *et al.*, 2013). Similar to the earlier observation by Liebrand *et al*. (2013), SOBIR1‐Myc does not co‐purify with FLS2‐eGFP, here used as a negative control (Fig. 2A).

A split‐YFP (yellow fluorescent protein) experiment confirmed the homodimerization of *Sl*SOBIR1 *in planta* (Fig. 2B). For this, *Sl*SOBIR1 fused to the N‐terminal half of YFP (nYFP) was co‐expressed with *Sl*SOBIR1 fused to the C‐terminal half of YFP (cYFP). Subsequent observation by confocal microscopy revealed a clear signal of reconstituted full‐length YFP. As a positive control, co‐expression of *Sl*SOBIR1‐nYFP with Cf‐4‐cYFP was performed, which also resulted in a clear YFP signal. The expression of *Sl*SOBIR1‐nYFP in combination with *At*FLS2‐cYFP, performed as a negative control, did not reconstitute a YFP signal (Fig. 2B). A clear YFP signal on co‐expression of *At*FLS2‐cYFP and *At*FLS2‐nYFP (Fig. 2B) confirmed that FLS2 also forms homodimers *in planta* (Sun *et al.*, 2012).

As *At*SOBIR1 is phosphorylated *in planta *(Fig. 1A), and SOBIR1 is able to constitutively form homodimers *in planta*, it is mechanistically possible that SOBIR1 trans‐autophosphorylates.

### Constitutive immune activity of *At*SOBIR1 is dependent on BAK1

**Figure 3:**
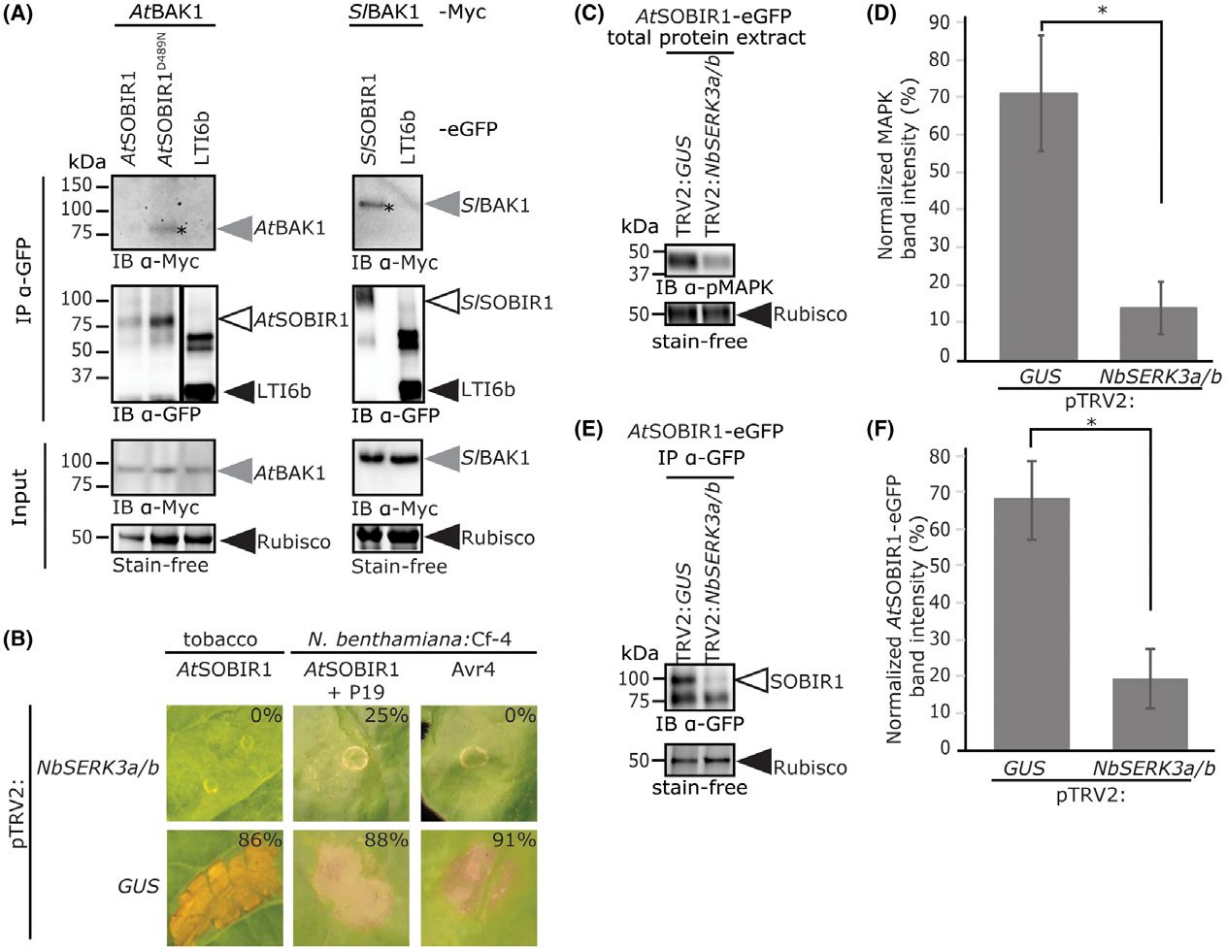
Constitutive immune activity of *At*SOBIR1 is dependent on BAK1. (A) SOBIR1 constitutively interacts with BAK1. eGFP‐tagged versions of SOBIR1 and Myc‐tagged versions of BAK1 were transiently co‐expressed in *N. benthamiana* at an OD_600_ of 0.6 for each construct. Leaves were harvested at 1 dpi, as *At*SOBIR1 triggers a constitutive cell death visible at 2 dpi, followed by IP and protein detection on IB. It should be noted that anti‐Myc IB, revealing the co‐IP of BAK1‐Myc (asterisks), shows relatively faint signals, which suggests that only a small pool of the total amount of SOBIR1 and BAK1 protein constitutively interacts. The plasma membrane (PM) protein LOW TEMPERATURE‐INDUCED 6b (LTI6b) (Kurup et al., 2005), included as a negative control, is visible at 30 kDa. The band at 60 kDa could be a dimer of LTI6b that is not fully denatured. The representative results of three independent experiments are shown. (B) Inoculation of tobacco and *N. benthamiana:Cf‐4* with pTRV2:*NbSERK3a/b* leads to compromised constitutive immune activity of *At*SOBIR1. *At*SOBIR1‐eGFP was transiently expressed by agroinfiltration at an OD_600_ of 1 in pTRV2:*GUS*‐ or pTRV2:*NbSERK3a/b*‐inoculated tobacco or *N. benthamiana:Cf‐4*. In *N. benthamiana:Cf‐4*, *At*SOBIR1 was co‐expressed with P19 at an OD_600_ of 1. Transient expression of Avr4 at OD_600_ = 0.03 shows a compromised hypersensitive response (HR) in pTRV2:*NbSERK3a/b*‐inoculated *N. benthamiana:Cf‐4* plants. The percentages of cell death were scored as described in Experimental procedures. The experiments were performed at least three times, with testing of at least three individual leaves per sample. Representative photographs taken at 3 dpi are shown. (C) Inoculation with pTRV2:*NbSERK3a/b* leads to reduced mitogen‐activated protein kinase (MAPK) activation on transient overexpression of *At*SOBIR1. *At*SOBIR1‐eGFP was transiently co‐expressed with P19, both at an OD_600_ of 1, in *N. benthamiana *plants that had been previously inoculated with the indicated TRV recombinants. About 40 h after agroinfiltration, total protein was extracted and analysed for MAPK activation using anti‐p42/p44‐erk antibody. The Rubisco band shows equal loading. The experiment was performed three times, and representative results are shown. (D) Quantification of the results of the experiment shown in (C). Ratios were obtained by dividing the band intensity of phosphorylated MAPK by the intensity of the Rubisco input band. Data are presented as mean ± standard error (SE). The asterisk indicates a significant difference (*P* < 0.05), as determined by Student’s *t*‐test. (E) Inoculation with pTRV2:*NbSERK3a/b* leads to reduced accumulation of *At*SOBIR1‐eGFP. *At*SOBIR1‐eGFP was transiently co‐expressed with P19, both at an OD_600_ of 1, in *N. benthamiana *plants that had been previously inoculated with the indicated TRV recombinants. About 40 h after agroinfiltration, total protein was extracted and subjected to IP, followed by protein detection on IB. The Rubisco background band of the IP samples is depicted to show equal loading. The experiment was performed three times, and representative results are shown. (F) Quantification of the results of the experiment shown in (D). Ratios were obtained by dividing the band intensity of *At*SOBIR1‐eGFP by the band intensity of the Rubisco background in the IP samples. Data are presented as the mean ± SE. The asterisk indicates a significant difference (*P* < 0.05), as determined by Student’s *t*‐test. [Colour figure can be viewed at wileyonlinelibrary.com]

To analyse whether BAK1, in addition to its role in RLP/SOBIR1‐mediated immunity, also plays a role in *At*SOBIR1‐induced constitutive immunity and phosphorylation of SOBIR1, we examined whether, in addition to ligand‐induced BAK1 recruitment, a constitutive interaction between BAK1 and SOBIR1 takes place. To test this, we co‐expressed tagged BAK1 and SOBIR1 in *N. benthamiana* in the presence of P19. Interestingly, we found that BAK1 co‐immunoprecipitates with *At*SOBIR1^D489N^ and *Sl*SOBIR1 (Fig. 3A). Wild‐type *At*SOBIR1 often only accumulates to low levels because of its constitutive immune activity (Gao *et al.*, 2009; Wu *et al.*, 2018), and a complex of SOBIR1 and BAK1 is likely to be degraded by the plant to prevent the onset of an immune response and, consequently, cell death (Mbengue *et al.*, 2016). Therefore, the presence of a possible interaction between *At*BAK1 and *At*SOBIR1 could not be determined (Fig. 3A). The fact that we could not determine an interaction between *At*BAK1 and *At*SOBIR1 is in agreement with the findings of Liu *et al*. (2016), who showed an interaction between *At*SOBIR1 and *At*BAK1 only on silencing of *BIR1* in *Arabidopsis*. It is probable that a small pool of SOBIR1 and BAK1 constitutively interacts when overexpressed *in planta*, and this pool becomes larger on ligand elicitation (Albert *et al.*, 2015; Postma *et al.*, 2016; Wang *et al.*, 2018) or BIR1 silencing (Liu *et al.*, 2016). Together, these results suggest that SOBIR1 forms heterodimers with BAK1, independent of the constitutive immune activity of SOBIR1, and the strong phosphorylation status of constitutively active *At*SOBIR1 cannot simply be explained by SOBIR1 interaction with BAK1, as *Sl*SOBIR1 also constitutively interacts with BAK1 and phosphorylation of *Sl*SOBIR1 is not apparent (Fig. 1A).

To further address whether BAK1 is required for *At*SOBIR1 constitutive immune activity, we employed virus‐induced gene silencing (VIGS) to knock down the expression of the *AtBAK1* homologues *SERK3a/b* and *SERK1* in tobacco and *N. benthamiana* (Heese *et al.*, 2007; Postma *et al.*, 2016). For this, the VIGS construct pTRV2:*NbSERK3a/b *(Heese *et al.*, 2007) was agroinoculated into tobacco (cv. Samsun) (Zhang *et al.*, 2013b), together with pTRV2:*GUS* as a negative control. Three weeks after agroinoculation, plants exhibited the characteristic stunting phenotype caused by silencing of the *BAK1 *homologues (Heese *et al.*, 2007; Li *et al.*, 2002; Nam and Li, 2002), and leaf sectors were transiently transformed to overexpress *At*SOBIR1. Constitutive immune activity of *At*SOBIR1 was highly compromised in plants inoculated with pTRV2:*NbSERK3a/b*, but not in pTRV2:*GUS*‐inoculated tobacco plants (Fig. 3B). A similar experiment was performed in *N. benthamiana:Cf‐4 *(Gabriëls *et al.*, 2006), for which inoculation with pTRV2:*NbSERK3a/b *also resulted in severe stunting, confirming *BAK1* silencing (Heese *et al.*, 2007; Li *et al.*, 2002; Nam and Li, 2002), and suppressed *At*SOBIR1 constitutive immune activity (Fig. 3B). Compromised Cf‐4‐mediated HR on expression of Avr4 in these plants confirmed the successful knockdown of *NbSERK3a/b*, as these BAK1 homologues have been shown previously to be required for Cf‐4/Avr4‐triggered HR (Postma *et al.*, 2016).

To confirm that the compromised cell death response, observed on overexpression of *At*SOBIR1 in *N. benthamiana* inoculated with pTRV2:*NbSERK3a/b*, is a consequence of reduced constitutive immune activity, we examined MAPK activation. MAPKs are constitutively activated on overexpression of *At*SOBIR1 (Wu *et al.*, 2018), and immune blotting (IB) showed that MAPK activation by overexpression of *At*SOBIR1 is reduced in plants inoculated with pTRV2:*NbSERK3a/b*, but not in pTRV2:*GUS*‐inoculated plants (Fig. 3A,C).

Previously, it has been shown that the accumulation levels of *At*SOBIR1 variants inversely correlate with their constitutive immune activity (Wu *et al.*, 2018). Interestingly, although showing less constitutive immune activity, *At*SOBIR1 accumulates to lower levels in leaves of pTRV2:*NbSERK3a/b*‐inoculated *N. benthamiana* plants, when compared with pTRV2:*GUS*‐inoculated plants, in which *At*SOBIR1 shows strong immune activity. This suggests that these BAK1 homologues are also important for the accumulation of SOBIR1 (Fig. 3E,F). Therefore, the reduced accumulation of *At*SOBIR1 might also partially explain the reduced immune phenotypes.

Together, these experiments show that SOBIR1 forms heterodimers with BAK1, and that SOBIR1 constitutive immunity and accumulation are dependent on BAK1.

### SOBIR1‐mediated immunity is dependent on kinase‐active BAK1

**Figure 4:**
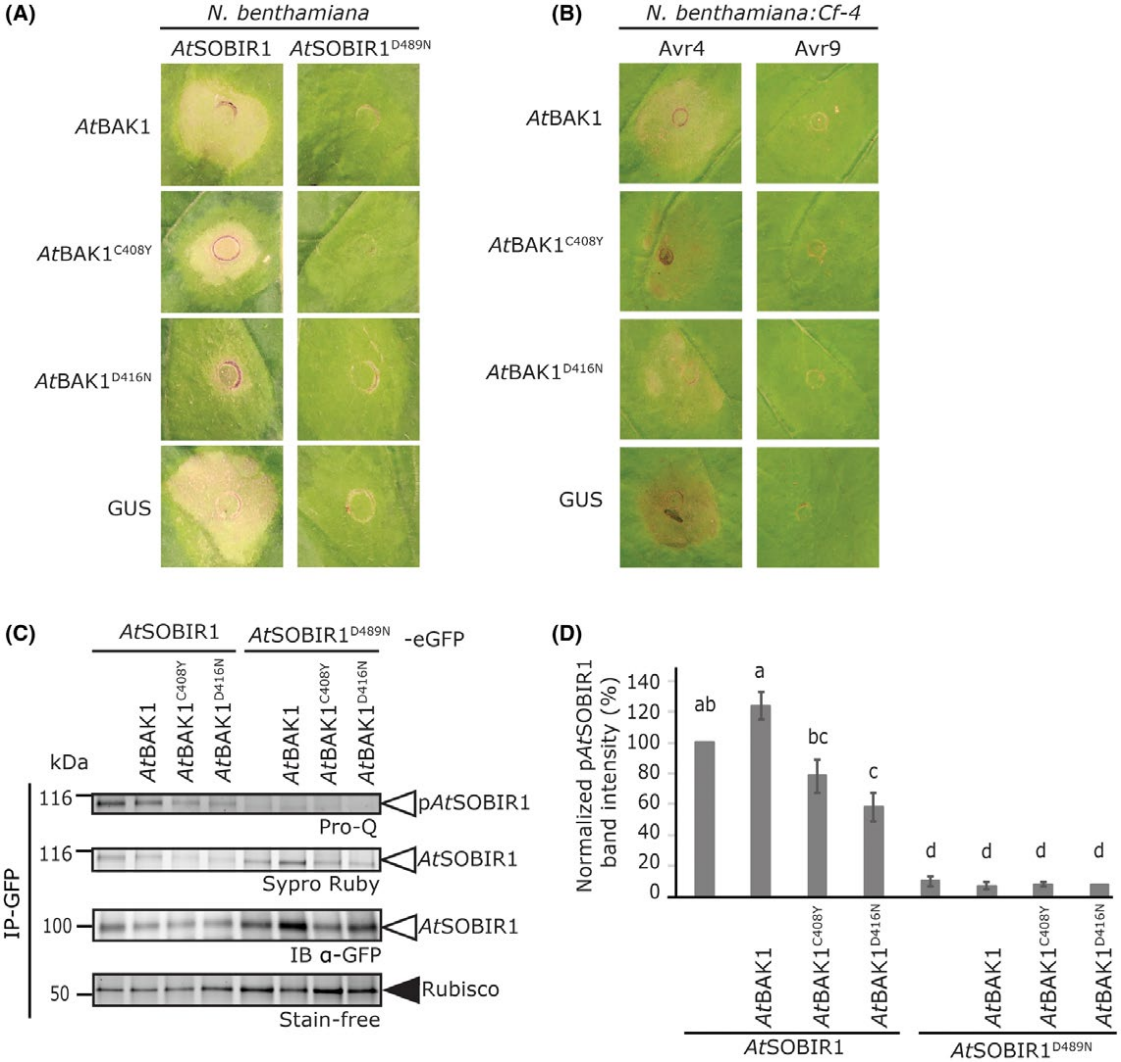
SOBIR1‐mediated immunity is dependent on kinase‐active BAK1. (A) Transient co‐expression of *At*SOBIR1 with the *At*BAK1^C408Y^ or *At*BAK1^D416N^ mutant results in reduced *At*SOBIR1 constitutive immune activity, when compared with co‐expression with wild‐type *At*BAK1 or β‐glucuronidase (GUS). The indicated constructs were agroinfiltrated in *N. benthamiana* at an OD_600_ of 0.5, with co‐infiltration of P19 at an OD_600_ of 1. Photographs were taken at 3 dpi, and are representative of two repetitions, with eight agroinfiltrated leaves per sample. (See also Fig. S3A,B.) (B) Transient co‐expression of Avr4 in *N. benthamiana:Cf‐4* with the mutants *At*BAK1^C408Y^ or *At*BAK1^D416N^ results in reduced Cf‐4/Avr4 HR, when compared with co‐expression with wild‐type *At*BAK1 or GUS*.* The indicated constructs were agroinfiltrated at an OD_600_ of 0.5, with co‐infiltration of Avr4 or Avr9 at an OD_600_ of 0.03. Photographs were taken at 3 dpi, and are representative of three repetitions, with agroinfiltration of at least six leaves per sample. (See also Fig. S3C.) (C) Phosphorylation of *At*SOBIR1 is dependent on kinase‐active BAK1. The indicated eGFP‐tagged *At*SOBIR1 and untagged BAK1 constructs were co‐agroinfiltrated at an OD_600_ of 0.5, in combination with P19 at an OD_600_ of 1. At 2 dpi, leaves were harvested and subjected to immunoprecipitation (IP) using GFP‐affinity beads, and proteins were subsequently detected by anti‐GFP immune blotting (IB) and Pro‐Q stain. The ribulose‐1,5‐bisphosphate carboxylase/oxygenase (Rubisco) background band of the IP samples is depicted to show equal loading. The experiment was performed twice, and representative results are shown. (D) Quantification of the band intensity of phosphorylated *At*SOBIR1 as shown in (C). Ratios were obtained by dividing the band intensity of p*At*SOBIR1 from Pro‐Q staining by the band intensity reflecting the total amount of immunoprecipitated *At*SOBIR1 in Sypro Ruby staining. Data are presented as the mean ± standard error (SE). The band intensity of the control sample, expression of *At*SOBIR1 without co‐expression of BAK1, was set at 100%. The letters indicate significant differences at *P* < 0.05, as determined by one‐way analysis of variance (ANOVA), including a Tukey *post hoc* test. [Colour figure can be viewed at wileyonlinelibrary.com]

To further elucidate by which mechanism(s) BAK1 regulates the constitutive immune activity of *At*SOBIR1, transient co‐transformations of *At*SOBIR1 with (untagged) BAK1 variants, mutated in their kinase domain, were performed. These experiments revealed a dominant‐negative effect of the BAK1 mutant *At*BAK1^C408Y^ (also known as *At*BAK1‐5) and kinase‐dead *At*BAK1^D416N ^(Schwessinger *et al.*, 2011) in the form of suppression of *At*SOBIR1 constitutive immune activity in both *N. benthamiana* and tobacco (Figs 4A and S3A,B, see Supporting Information). Similarly, the Cf‐4/Avr4‐induced HR was suppressed by co‐expression of *At*BAK1^C408Y^ and *At*BAK1^D416N^ with Avr4 in *N. benthamiana:Cf‐4* plants (Figs 4B and S3C). *At*BAK1^C408Y^ is known to compromise immunity mediated by FLS2 and EFR, but does not affect BR signalling or cell death induction (Schwessinger *et al.*, 2011). *At*BAK1^D416N^ exhibits a broad loss‐of‐function phenotype, as this mutant has lost its ability to signal in FLS2‐ and EFR‐mediated immune signalling, as well as in BR and cell death signalling (Schwessinger *et al.*, 2011). Our results suggest that the transiently overexpressed *At*BAK1 mutants out‐compete endogenous functional BAK1 homologues, resulting in compromised *At*SOBIR1 constitutive immunity. These findings show that the constitutive immunity of *At*SOBIR1 is dependent on defence signalling‐competent BAK1.

To determine whether co‐expression of the signalling‐incompetent BAK1 mutants affects the phosphorylation status of *At*SOBIR1, we co‐agroinfiltrated *At*SOBIR1‐eGFP with either wild‐type BAK1 or the different BAK1 mutants in *N. benthamiana*, in the presence of P19. Subsequent IP of *At*SOBIR1, followed by Pro‐Q staining, revealed a reduction in the phosphorylation level of *At*SOBIR1 when co‐expressed with *At*BAK1^C408Y^ or *At*BAK1^D416N^, when compared with its co‐expression with wild‐type BAK1 (Fig. 4C,D). This analysis suggests that the strong phosphorylation of *At*SOBIR1 is probably the result of transphosphorylation of *At*SOBIR1 by BAK1.

Taken together, these data show that the elevated phosphorylation status of constitutively active SOBIR1 depends on signalling‐competent BAK1. This suggests that immune signalling by SOBIR1 involves transphosphorylation events with BAK1.

## Discussion

**Figure 5:**
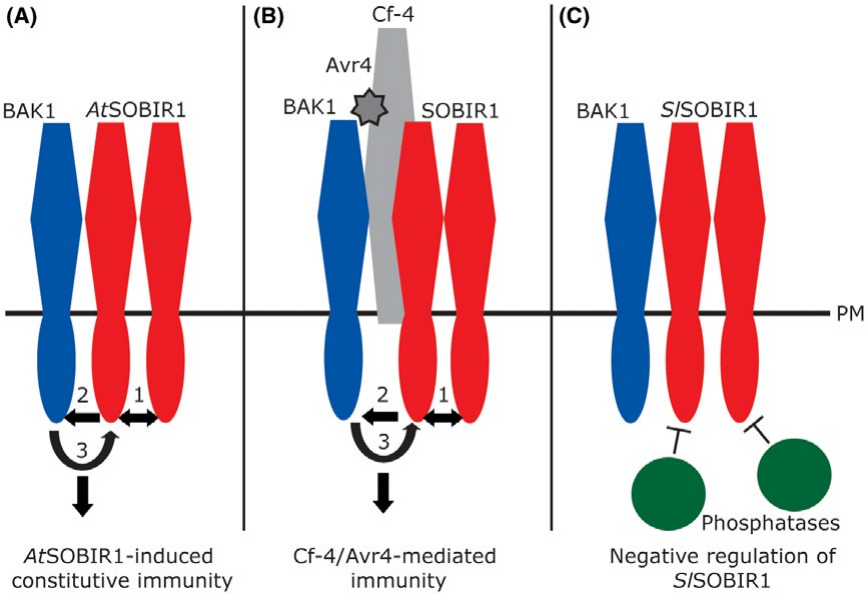
SOBIR1 and BAK1 act together to signal for defence. In this model, we propose that SOBIR1 and BAK1 act together to signal for downstream defence activation. In addition to the trans‐autophosphorylation of *At*SOBIR1, it is likely that *At*SOBIR1 transphosphorylates BAK1. By transphosphorylating BAK1, this co‐receptor becomes activated, followed by the transphosphorylation and activation of *At*SOBIR1 by activated BAK1 to enable *At*SOBIR1‐induced constitutive immunity (A) and the Cf‐4/Avr4‐mediated HR (B). The arrows represent auto‐ or transphosphorylation events, and the numbers indicate the proposed order of the various phosphorylation events. The phosphorylation and activity of *Solanum lycopersicum* (*Sl*)SOBIR1 are likely to be down‐regulated by endogenous phosphatases, when not activated by Avr4 (C). PM, plasma membrane. [Colour figure can be viewed at wileyonlinelibrary.com]

BAK1 is a well‐known co‐receptor for ligand‐binding RLKs, such as BRI1, EFR and FLS2 (Chinchilla *et al.*, 2009), and has been found recently to be recruited to activated RLP/SOBIR1 complexes, which have been proposed to function as two‐component RLKs (Albert *et al.*, 2015; Domazakis *et al*., 2018; Liebrand *et al.*, 2014; Postma *et al.*, 2016; Wang *et al.*, 2018). Here, we show that the kinase activity of *At*SOBIR1 is essential for its phosphorylation. In addition, we show that SOBIR1 and BAK1 act together to signal for defence. Interestingly, in addition to kinase‐active SOBIR1, kinase‐active BAK1 is essential for *At*SOBIR1 constitutive immune activity, for strong phosphorylation of *At*SOBIR1 and for the Cf‐4/Avr4‐triggered HR. Based on our findings and on current data concerning RLKs, such as FLS2, which recruit and transphosphorylate BAK1 on ligand binding (Schwessinger *et al.*, 2011; Somssich *et al.*, 2015; Wang *et al.*, 2008; Yan *et al.*, 2012), we speculate that SOBIR1/RLP bimolecular RLKs function in a similar way when in a complex with BAK1. We propose a model in which *At*SOBIR1 trans‐autophosphorylates and transphosphorylates BAK1, thereby activating this co‐receptor (Fig. 5). Subsequent transphosphorylation and full activation of *At*SOBIR1 by activated BAK1 probably enables *At*SOBIR1‐induced constitutive immunity (Fig. 5A). The model also applies to Cf‐4/Avr4‐mediated immunity, where the recognition of Avr4 by Cf‐4 possibly initiates SOBIR1 trans‐autophosphorylation, followed by transphosphorylation of BAK1 by SOBIR1, and, in turn, transphosphorylation of SOBIR1 by activated BAK1 to fully activate SOBIR1 (Fig. 5B). Fully activated SOBIR1 subsequently initiates further downstream signalling.

First, we found that the phosphorylation status of SOBIR1 and its constitutive immune activity are positively linked, as constitutively active *At*SOBIR1 is clearly phosphorylated, and phosphorylation of kinase‐dead *At*SOBIR1 is not apparent (Fig. 1). In addition, we observed suppressed phosphorylation of *At*SOBIR1 when co‐expressed with signalling‐incompetent BAK1 mutants (Fig. 4). This suggests that BAK1 plays a role in the transphosphorylation and activation of *At*SOBIR1. Knockdown of the expression of *BAK1 *homologues *NbSERK1 *and* NbSERK3a/b* in *N. benthamiana* causes *At*SOBIR1 to accumulate to lower levels (Fig. 3E), which might also partially explain the compromised *At*SOBIR1‐induced constitutive immunity in these *NbSERK3a/b*‐silenced plants. Stabilization of RLKs and RLPs by regulatory RLKs has been observed previously, and so it is likely that SOBIR1 and BAK1 promote each other’s accumulation (Imkampe *et al.*, 2017; Liebrand *et al.*, 2013; Wang *et al.*, 2008). Because silencing of *BAK1* homologues suppresses the accumulation of SOBIR1, attempts to measure changes in the phosphorylation level of SOBIR1 on silencing of *BAK1* homologues were not successful.

A constitutive interaction between SOBIR1 and BAK1 has been suggested previously (Liu *et al.*, 2016; Postma *et al.*, 2016). Although Liu *et al.* (2016) did not show an interaction between *At*SOBIR1 and *At*BAK1 in *N. benthamiana*, they did so in *Arabidopsis* on silencing of a negative regulator of defence, *AtBIR1 *(Gao *et al.*, 2009; Liu *et al.*, 2016)*.* Silencing of *BIR1* led to higher BAK1 availability, and a clearer interaction of BAK1 with SOBIR1 (Liu *et al.*, 2016). In the experiment described here, the accumulation of *At*SOBIR1 is relatively low because of its constitutive immune activity in *N. benthamiana* (Wu *et al.*, 2018), and an interaction between *At*BAK1 and *At*SOBIR1 is not visible (Fig. 3A). For *At*SOBIR1^D489N^ and *Sl*SOBIR1, we observed an interaction with BAK1, because these SOBIR1 variants accumulate to higher amounts as they do not trigger a constitutive cell death response. A constitutive interaction between SOBIR1 and BAK1 is anticipated to occur only at very low levels, as this interaction is specifically stimulated on ligand perception by the SOBIR1‐associated RLP, in order to activate the immune system (Albert *et al.*, 2015; Domazakis *et al*., 2018; Postma *et al.*, 2016; Wang *et al.*, 2018). Pre‐formation of immune complexes at nanoclusters at the PM, without their activation by the matching ligand, has been shown previously (Bücherl *et al.*, 2017; Jarsch *et al.*, 2014; Somssich *et al.*, 2015). It was found that various pre‐formed immune complexes are present at the PM, spatially separated into different nanoclusters. Such a separation into different nanodomains enables rapid and diverse responses. Thus, a small pool of pre‐formed RLP/SOBIR1/BAK1 complexes, probably kept in check by endogenous phosphatases as suggested previously (Wu *et al.*, 2018), might enable rapid and specific responses to elicitation in resistant plants.

In addition to the sequestration of co‐receptors required for downstream signalling, immune receptor activity is also regulated at the level of phosphorylation (Couto and Zipfel, 2016). For instance, BAK1 and FLS2 are negatively regulated by PP2A and PP2C, respectively (Couto *et al*., 2016; Gómez‐Gómez *et al.*, 2001; Segonzac *et al.*, 2014). Here, we show that *At*SOBIR1, which can constitutively activate immune responses, is clearly phosphorylated when overexpressed in *N. benthamiana*, whereas *Sl*SOBIR1 is not (Fig. 1A). It is likely that endogenous phosphatases of *N. benthamiana* keep *Sl*SOBIR1 in check by dephosphorylation (Fig. 5C), but do not have the correct affinity for the phosphorylated kinase domain of *At*SOBIR1, and therefore cannot properly dephosphorylate this non‐solanaceous protein. This could lead to excessive phosphorylation and thereby constitutive immune activation, as suggested previously (Wu *et al.*, 2018).

Similar to BAK1, SOBIR1 is a typical RD‐kinase as it contains an arginine (R) and aspartic acid (D) residue in its catalytic site. RD‐kinases are generally thought to require phosphorylation of their activation loop to acquire the active conformation (Johnson *et al.*, 1996; Kornev *et al.*, 2006; Nolen *et al.*, 2004). Our observation that kinase‐dead *At*SOBIR1^D489N^ does not show substantial phosphorylation, when compared with wild‐type *At*SOBIR1, when transiently expressed in *N. benthamiana*, suggests that *At*SOBIR1 needs to trans‐autophosphorylate to a certain level in order to become signalling competent. As SOBIR1 forms homodimers, it is possible that such trans‐autophosphorylation takes place (Fig. 2). This trans‐autophosphorylation is probably required to keep SOBIR1 in a signalling‐active state. In addition, the lack of apparent phosphorylation of *At*SOBIR1^D489N ^suggests that *At*SOBIR1 needs to transphosphorylate its signalling partner BAK1 in order for this partner to become signalling competent, which then, in turn, transphosphorylates *At*SOBIR1. Indeed, on overexpression of BAK1 together with SOBIR1, we observed elevated phosphorylation levels of SOBIR1 (Fig. 4C, D). Moreover, overexpression of *At*SOBIR1 with signalling‐incompetent BAK1 mutants, which are unable to signal for defence, resulted in reduced phosphorylation levels of SOBIR1. These observations suggest that, for full phosphorylation of SOBIR1, and immune signalling by SOBIR1, transphosphorylation by BAK1 is probably necessary. This is in concert with previous studies on ligand binding RLKs and BAK1, for which it was concluded that transphosphorylation of RLK and BAK1 is necessary to initiate signalling on ligand perception (Wang *et al.*, 2008; Yan *et al.*, 2012). It is likely that, on ligand recognition by an RLP, the RLP and associated SOBIR1 undergo conformational changes, leading to SOBIR1 trans‐autophosphorylation, thereby providing SOBIR1 with the ability to trans‐phosphorylate BAK1, which is recruited on ligand perception by the RLP. This transphosphorylation event locks BAK1 in the active conformation (Yan *et al.*, 2012), and provides BAK1 with the capacity to transphosphorylate and fully activate SOBIR1. The resulting activated complex is subsequently able to trigger downstream defence signalling (Fig. 5).

Interestingly, we observed reduced *At*SOBIR1 phosphorylation levels and cell death on overexpression of *At*BAK1^C408Y^ (also referred to as BAK1‐5) together with *At*SOBIR1 (Fig. 4). *At*BAK1^C408Y ^was initially found to be impaired in complementing defence signalling by FLS2 and EFR (Schwessinger *et al.*, 2011). Nevertheless, *At*BAK1^C408Y^ exhibits only slightly reduced kinase activity, and is not impaired in BR or cell death signalling. Therefore, this mutant is important to unlink the ability of BAK1 to signal for defence, cell death and development. We observed that *At*BAK1^C408Y^ is impaired in signalling for *At*SOBIR1‐induced constitutive immunity, as well as in mediating the transphosphorylation of *At*SOBIR1 (Fig. 4). Thus, *At*SOBIR1 specifically signals for immunity together with BAK1. This further confirms that the constitutive immune activity induced by *At*SOBIR1 follows the same immune pathway as Cf‐4/Avr4‐mediated HR.

In conclusion, we have shown that SOBIR1 and BAK1 probably act together to signal for defence. Our *in planta *data support a model in which SOBIR1 trans‐autophosphorylates, and on elicitation transphosphorylates BAK1, and is, in turn, transphosphorylated by activated BAK1 to signal for immunity. We envisage that this model not only describes signalling for *At*SOBIR1 constitutive immunity, but also signalling on BAK1 heterodimer formation with ligand‐activated bipartite RLP/SOBIR1 complexes (Fig. 5).

## Experimental Procedures

### Binary vectors for *Agrobacterium*‐mediated transformation and VIGS

The constructs pBIN‐KS‐35S::*At*SOBIR1‐eGFP, pBIN‐KS‐35S::*At*SOBIR1^D489N^‐eGFP, pBIN‐KS‐35S::*Sl*SOBIR1‐eGFP, pBIN‐KS‐35S::*Sl*SOBIR1^D486N^‐eGFP, pBIN‐KS‐35S::*Sl*Cf‐4‐eGFP, pGWB20‐35S::*Sl*SERK3‐myc, pGWB20‐35s::*Sl*SOBIR1‐Myc and pGWB20‐35s::*At*SOBIR1‐Myc have been described previously (Liebrand *et al.*, 2012, 2013). *At*BAK1‐Myc has been described by Halter *et al*. (2014b). Avr4 was expressed using the pMOG800 construct (Van der Hoorn *et al.*, 2000). P19 (Voinnet *et al.*, 2015), pBIN61‐GUS, pTRV1 (Liu *et al.*, 2002,b), pTRV2:*GUS* (Tameling and Baulcombe, 2007) and pTRV2:*NbSERK3a/b* (Heese *et al.*, 2007) have been described elsewhere. *At*BAK1, *At*BAK1^C408Y^ and *At*BAK1^D416N^ originate from Schwessinger *et al. *(2011). GFP‐LTI6b has been described previously (Kurup *et al.*, 2005). *At*FLS2‐eGFP and *Sl*FLS2‐eGFP were described by Robatzek *et al. *(2006, 2007). *Sl*SOBIR1‐cYFP, *Sl*SOBIR1‐nYFP and *Sl*Cf‐4‐cYFP (Postma *et al.*, 2016), and pACA8::ACA8‐mCherry, *At*FLS2‐cYFP and *At*FLS2‐nYFP (Frei dit Frey *et al.*, 2012), have been described elsewhere.

### Plant growth conditions

*Nicotiana tabacum* (tobacco) (cv. SR1 and cv. Samsun) and *N. benthamiana* [wild‐type and *N. benthamiana* stably expressing *SlCf‐4 *under its native promoter (referred to as *N. benthamiana:Cf‐4*; Gabriëls *et al.*, 2006] were grown under 16 h light at 25 °C and 8 h darkness at 21 °C, with ~75% relative humidity.

### VIGS in tobacco and *N. benthamiana*

VIGS using TRV‐based vectors was performed in tobacco (cv. Samsun) and *N. benthamiana:Cf‐4* as described previously (Liebrand *et al.*, 2012; Zhang *et al.*, 2013b).

### *Agrobacterium*‐mediated transient transformation

*Agrobacterium*‐mediated transient transformations (agroinfiltrations) were performed as described previously (Van der Hoorn *et al.*, 2000). Binary constructs expressing affinity‐tagged proteins were agroinfiltrated with *Agrobacterium tumefaciens* cultures at an OD_600_ of 1 in combination with P19 at an OD_600_ of 1, unless indicated otherwise. Leaves were harvested for protein isolation and IP at 2 dpi, unless indicated otherwise. Percentages of HR were quantified by visual scoring for full HR (100%), mildly reduced HR (60%), strongly reduced HR (30%) and no HR (0%).

### IPs, IB, phosphorylation analysis and MAPK activation analysis

IPs and co‐IPs were performed as described previously (Liebrand *et al.*, 2013). To detect phosphorylated proteins, a protein extraction buffer was used as described by Karlova *et al*. (2006, 2009), with minor modifications; instead of Tris and Triton‐X, 100 mm NaPi (pH 7.2) and 1% IGEPAL CA‐630 (NP40) were used, respectively. Pre‐cast TGX gels were used for Pro‐Q and Sypro Ruby analyses (Bio‐Rad, Veenendaal, the Netherlands, #456‐1095). Pro‐Q diamond phosphoprotein gel stains and subsequent Sypro Ruby stains were performed according to the manufacturer’s recommendations (Invitrogen, Life Technologies, Carlsbad, CA, USA; Taylor *et al.*, 2013). TGX stain‐free gels were used for all other protein analyses (Bio‐Rad, #456‐8085), and total protein was visualized using the stain‐free method or with Coomassie Brilliant Blue (CBB). The following antibodies were used for protein detection on IB: αGFP‐HRP (130‐091‐833, MACS antibodies, Bergisch Gladbach, Germany), αMyc (cMyc9E10, sc‐40, Santa Cruz Biotechnology, Heidelberg, Germany), αMouse‐HRP (GE Healthcare, Eindhoven, The Netherlands), anti‐p42/p44‐erk (NEB: Bioké Dellaertweg 9b 2316 WZ, Leiden) and goat anti‐rabbit (Sigma Zwijndrecht, the Netherlands). Band intensities were measured using Image Lab software (Bio‐Rad), and ratios were calculated as indicated in the figures. To quantify immunoprecipitated protein bands, the ribulose‐1,5‐bisphosphate carboxylase/oxygenase (Rubisco) background band in the IP sample was taken as an internal standard for the total protein concentration of the sample.

### Protein localization studies

Confocal laser scanning microscopy (CLSM) was performed using a Leica SP5 laser point scanning microscope (Leica Camera AG, Wetzlar, Germany), mounted with hybrid detectors (HyD), as described previously (Beck *et al.*, 2012). For CLSM analysis of eGFP, constructs were transiently expressed in adult tobacco plants by infiltration with *Agrobacterium tumefaciens* suspensions of OD_600_ = 0.3. At 2 dpi, GFP fluorophores were excited using a 488‐nm argon laser and fluorescence emission was captured between 495 and 540 nm. mCherry fluorophores were excited using a 561‐nm argon laser and fluorescence emission was captured between 580 and 620 nm. For GFP‐only images, chloroplast autofluorescence was captured between 700 and 800 nm. For CLSM analysis of bimolecular fluorescence complementation (BiFC, split‐YFP) experiments, cYFP and nYFP constructs were transiently co‐expressed by co‐infiltration of adult *N. benthamiana* plants using *Agrobacterium tumefaciens* suspensions, each at OD_600_ = 0.3. Reconstituted YFP molecules were excited using a 514‐nm argon laser, and fluorescence emission was captured between 520 and 550 nm. Chloroplast autofluorescence was captured between 700 and 800 nm. Images were taken using a 20× objective (for eGFP) or 40× objective (for YFP), and processed using Leica LAS‐AF and FIJI (ImageJ) software packages.

## Supporting information


**Fig. S1  **Kinase activity of SOBIR1 is not required for Cf‐4 stabilization. (A) Transient overexpression of *Arabidopsis thaliana* (*At*)SOBIR1 induces cell death in tobacco and in *Nicotiana benthamiana* when co‐expressed with P19. Agroinfiltrations were performed at an optical density at 600 nm (OD_600_) of 1. Where indicated, P19 was also co‐infiltrated at an OD_600_ of 1. Photographs were taken at 3 days post‐infiltration (dpi). It should be noted that constitutive immune activity of *At*SOBIR1 requires its kinase activity. Furthermore, overexpression of *Solanum lycopersicum* (*Sl*)SOBIR1 from the Solanaceous plant tomato does not result in cell death. [See also Wu *et al.* (2018)]. (B) Co‐expression of wild‐type *Sl*SOBIR1 as well as kinase‐dead* Sl*SOBIR1^D473N^ stabilizes Cf‐4 when co‐expressed in *N. benthamiana*. It should be noted that the signal of Cf‐4 is increased when overexpressed with both wild‐type and kinase‐dead *Sl*SOBIR1, and highly increased on co‐expression with P19. Co‐agroinfiltrations of the affinity‐tagged proteins were performed in *N. benthamiana *leaves at an OD_600_ of 1 for each construct. Leaves were harvested at 2 dpi, and subjected to immunoprecipitation (IP) using anti‐green fluorescent protein (anti‐GFP) beads, followed by immune blotting (IB). The ribulose‐1,5‐bisphosphate carboxylase/oxygenase (Rubisco) band of the input shows equal loading. It should be noted that Cf‐4 is only visible in the input when co‐infiltrated with P19. CBB, Coomassie Brilliant Blue.

**Fig. S2  **Kinase‐dead SOBIR1 constitutively forms homodimers *in planta. *(A) Myc‐tagged versions of *At*SOBIR1^D489N^ and *Sl*SOBIR1^D473N^ co‐immunoprecipitate with eGFP‐tagged versions of *At*SOBIR1^D489N^ and *Sl*SOBIR1^D473N^ (asterisks), respectively, and with Cf‐4‐eGFP, but not with Flagellin‐Sensing 2 (FLS2)‐eGFP. Co‐agroinfiltrations of the various affinity‐tagged proteins were performed in combination with P19 in leaves of *N. benthamiana* at an OD_600_ of 0.6 for each construct. Leaves were harvested at 2 dpi, and subjected to IP using anti‐GFP beads, followed by IB. The Rubisco band of the input shows equal loading. It should be noted that, because of the low accumulation levels, not all proteins are visible in the input samples.

**Fig. S3** *At*SOBIR1‐mediated immunity is dependent on kinase‐active BAK1. (A) Transient co‐expression of *At*SOBIR1 in tobacco with *At*BAK1^C408Y^ or *At*BAK1^D416N^ results in reduced *At*SOBIR1 constitutive immune activity, when compared with co‐expression of *At*SOBIR1 with wild‐type *At*BAK1 or GUS. The indicated constructs were agroinfiltrated at an OD_600_ of 0.7. Photographs were taken at 2 dpi, and are representative of the agroinfiltration of eight leaves per sample. (B) Quantification of the percentage of cell death as shown in Fig. 4A. Percentages of constitutive cell death are presented as the mean ± standard error (SE). The letters indicate significant differences at *P* < 0.05, as determined by one‐way analysis of variance (ANOVA), including a Tukey *post hoc* test. (C) Quantification of the percentage of hypersensitive response (HR) as shown in Fig. 4B. Percentages of Avr4‐induced HR are presented as mean ± SE. The letters indicate significant differences at *P* < 0.05, as determined by one‐way ANOVA, including a Tukey *post hoc* test.
