# Supplementary figures and images for: Kinase activity of SOBIR1 and BAK1 is required for immune signalling
Source: Mol Plant Pathol. 2019 Jan 2;20(3):410–22. doi: 10.1111/mpp.12767 (PMC6637861; doi:10.1111/mpp.12767)

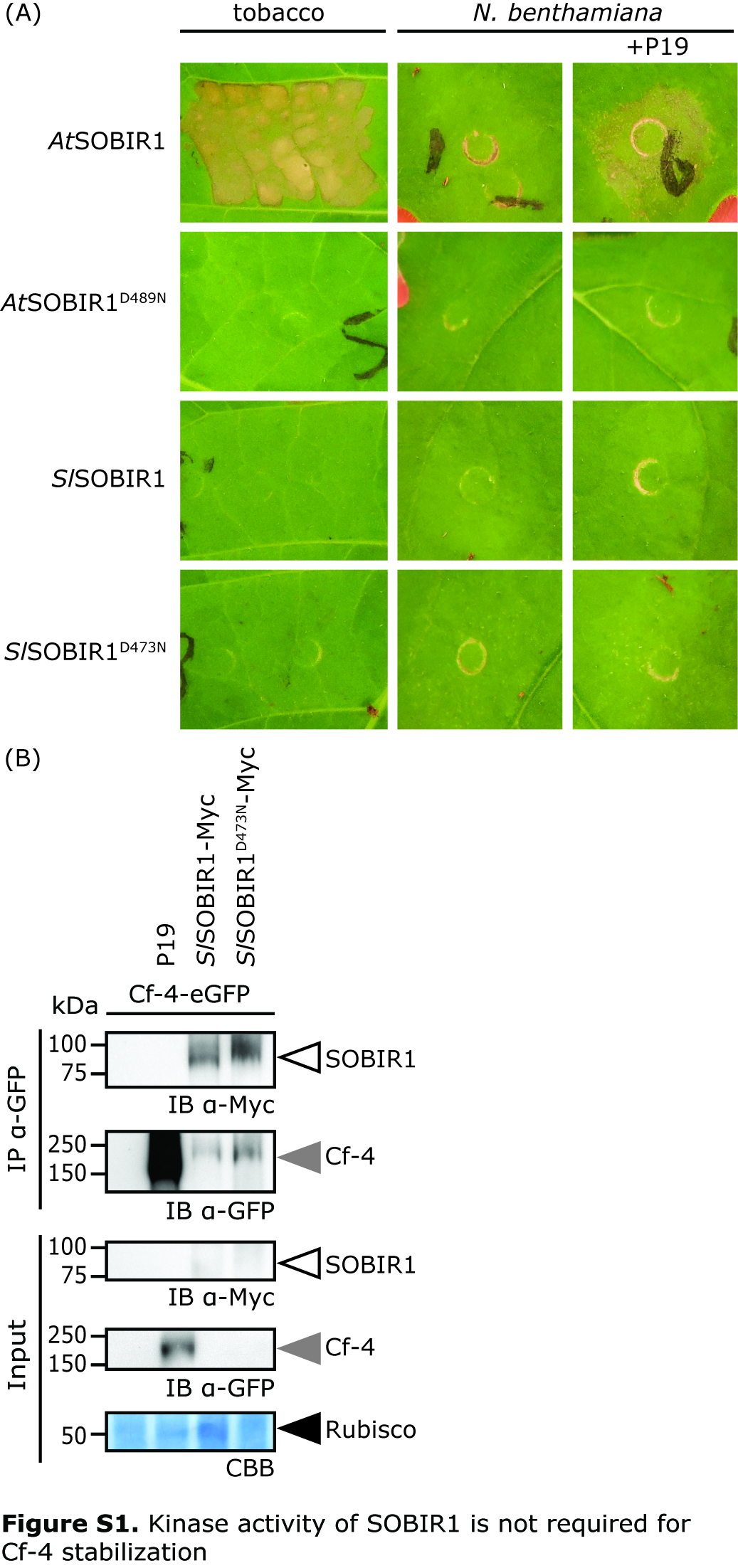

Supplement: Supplementary file 1 — Fig. S1 Kinase activity of SOBIR1 is not required for Cf‐4 stabilization. (A) Transient overexpression of Arabidopsis thaliana (At)SOBIR1 induces cell death in tobacco and in Nicotiana benthamiana when co‐expressed with P19. Agroinfiltrations were performed at an optical density at 600 nm (OD600) of 1. Where indicated, P19 was also co‐infiltrated at an OD600 of 1. Photographs were taken at 3 days post‐infiltration (dpi). It should be noted that constitutive immune activity of AtSOBIR1 requires its kinase activity. Furthermore, overexpression of Solanum lycopersicum (Sl)SOBIR1 from the Solanaceous plant tomato does not result in cell death. [See also Wu et al. (2018)]. (B) Co‐expression of wild‐type SlSOBIR1 as well as kinase‐dead SlSOBIR1D473N stabilizes Cf‐4 when co‐expressed in N. benthamiana. It should be noted that the signal of Cf‐4 is increased when overexpressed with both wild‐type and kinase‐dead SlSOBIR1, and highly increased on co‐expression with P19. Co‐agroinfiltrations of the affinity‐tagged proteins were performed in N. benthamiana leaves at an OD600 of 1 for each construct. Leaves were harvested at 2 dpi, and subjected to immunoprecipitation (IP) using anti‐green fluorescent protein (anti‐GFP) beads, followed by immune blotting (IB). The ribulose‐1,5‐bisphosphate carboxylase/oxygenase (Rubisco) band of the input shows equal loading. It should be noted that Cf‐4 is only visible in the input when co‐infiltrated with P19. CBB, Coomassie Brilliant Blue. [file MPP-20-410-s001.tif]

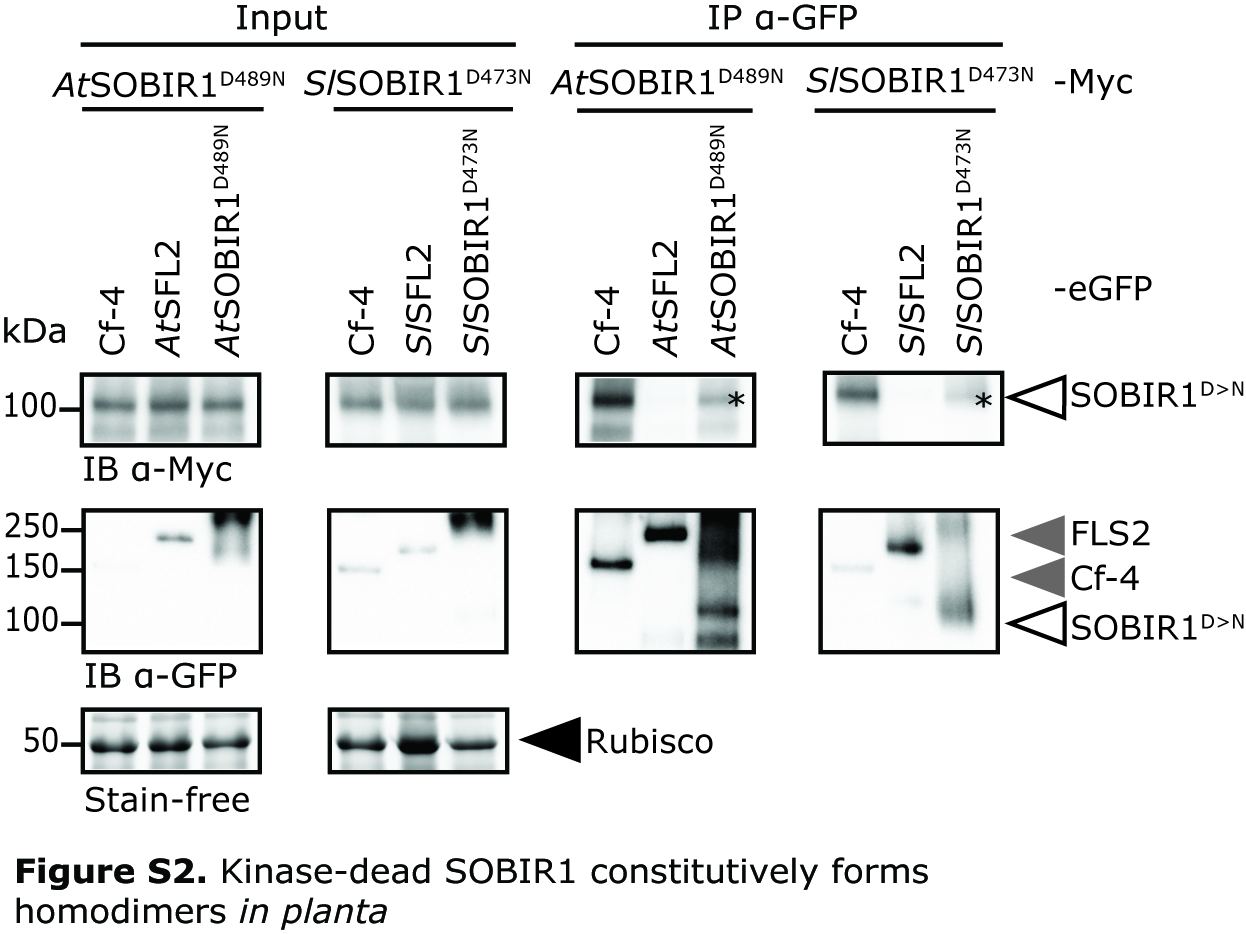

Supplement: Supplementary file 2 — Fig. S2 Kinase‐dead SOBIR1 constitutively forms homodimers in planta. (A) Myc‐tagged versions of AtSOBIR1D489N and SlSOBIR1D473N co‐immunoprecipitate with eGFP‐tagged versions of AtSOBIR1D489N and SlSOBIR1D473N (asterisks), respectively, and with Cf‐4‐eGFP, but not with Flagellin‐Sensing 2 (FLS2)‐eGFP. Co‐agroinfiltrations of the various affinity‐tagged proteins were performed in combination with P19 in leaves of N. benthamiana at an OD600 of 0.6 for each construct. Leaves were harvested at 2 dpi, and subjected to IP using anti‐GFP beads, followed by IB. The Rubisco band of the input shows equal loading. It should be noted that, because of the low accumulation levels, not all proteins are visible in the input samples. [file MPP-20-410-s002.tif]

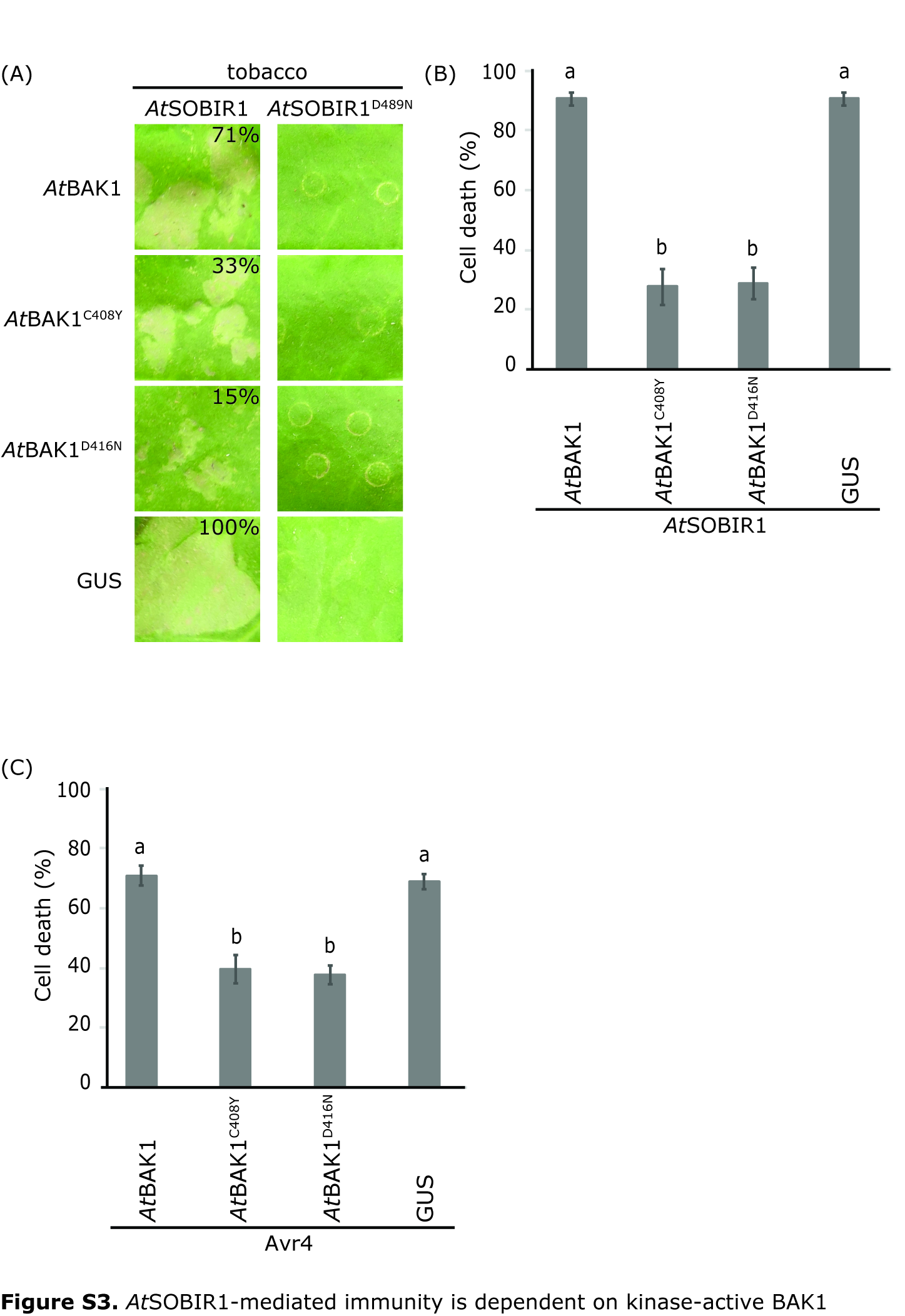

Supplement: Supplementary file 3 — Fig. S3 AtSOBIR1‐mediated immunity is dependent on kinase‐active BAK1. (A) Transient co‐expression of AtSOBIR1 in tobacco with AtBAK1C408Y or AtBAK1D416N results in reduced AtSOBIR1 constitutive immune activity, when compared with co‐expression of AtSOBIR1 with wild‐type AtBAK1 or GUS. The indicated constructs were agroinfiltrated at an OD600 of 0.7. Photographs were taken at 2 dpi, and are representative of the agroinfiltration of eight leaves per sample. (B) Quantification of the percentage of cell death as shown in Fig. 4A. Percentages of constitutive cell death are presented as the mean ± standard error (SE). The letters indicate significant differences at P < 0.05, as determined by one‐way analysis of variance (ANOVA), including a Tukey post hoc test. (C) Quantification of the percentage of hypersensitive response (HR) as shown in Fig. 4B. Percentages of Avr4‐induced HR are presented as mean ± SE. The letters indicate significant differences at P < 0.05, as determined by one‐way ANOVA, including a Tukey post hoc test. [file MPP-20-410-s003.tif]
